# Comparative analysis of cognitive behavioral therapy and dialectical behavior therapy in enhancing psychological capital among medical students: a randomized controlled trial

**DOI:** 10.3389/fpsyg.2024.1479310

**Published:** 2024-12-04

**Authors:** Cexin Dong, Jinfa Zhao, Yating Wei, Deyuan Wu, Zhu Cai

**Affiliations:** ^1^School of Mental Health and Psychological Science, Anhui Medical University, Hefei, China; ^2^School of Health Management, Anhui Medical University, Hefei, China

**Keywords:** psychological capital, medical students, group psychological intervention, group cognitive behavioral therapy, dialectical behavior therapy

## Abstract

**Background:**

Medical students are confronted with a complex stress environment, encompassing academic challenges, residency training, and future workplace pressures. Therefore, the exploration of effective psychological capital intervention strategies is crucial for enhancing their mental health and promoting career development.

**Objective:**

The aim of this study was to evaluate the efficacy of group cognitive-behavioral therapy (GCBT) and dialectical behavior therapy (DBT) in boosting the psychological capital of medical students and to compare the advantages and disadvantages of these two therapies.

**Method:**

A randomized controlled trial was conducted, recruiting 56 second-year medical students, who were randomly assigned into three groups: GCBT intervention group, DBT intervention group, and control group. The intervention period lasted for 6 weeks, during which the GCBT and DBT groups received respective interventions, while the control group did not receive any intervention.

**Results:**

Regardless of whether GCBT or DBT was used as an intervention, the psychological capital levels of the intervention group students showed significant improvement (mean ± SD, *p* < 0.05), with effect sizes ranging from 0.324 to 0.667. Further follow-up studies revealed that this improvement remained stable within 1 month post-intervention (mean ± SD, *p* < 0.05).

**Conclusion:**

Both group cognitive-behavioral therapy (GCBT) and dialectical behavior therapy (DBT) have been proven to be effective psychological intervention methods, capable of significantly enhancing the psychological capital of medical students. However, there are certain differences in their effects, providing diverse intervention options to cater to the varied psychological needs of medical students.

## 1 Introduction

During their training and academic years, as well as in their future careers, medical students must deal with a variety of stressors. These include, but are not limited to, the demands of rigorous coursework and exams, the need to develop operational skills and adjust to clinical settings, the difficulties of high-intensity work in the workplace, patient responsibility, and professional competition (Puthran et al., 2016; Gan and Yuen, 2019; Chen et al., 2022; Xu et al., 2023; Gao et al., 2022). Among medical students, this set of pressures can quickly lead to anxiety, despair, and other negative emotions, which can then develop into mental health issues. Given this, a thorough investigation and analysis of medical students’ mental health status is essential for identifying the causes and coping strategies of their psychological stress as well as for creating successful intervention plans that support their mental well-being and professional advancement.

Positive psychological traits, including optimism, hope, self-efficacy, and mental resilience, are all included in psychological capital (PsyCap), one measure of mental health. PsyCap has been demonstrated to improve subjective well-being, which in turn lowers stress and negative emotions, and it has a substantial effect on a person’s mental health and academic achievement (Yu et al., 2022). Improving psychological capital, particularly for students, can aid in stress relief, anxiety reduction, and depression reduction, supporting the improvement of mental health (Prasath et al., 2022; Wu et al., 2019). Considering the three unique pressures that medical students encounter—study, internship training, and employment—enhancing psychological capital is thought to be a successful approach to enhancing mental health.

Even while the value of psychological capital in medical education is becoming more well recognized, little research has been done on the subject, especially when it comes to comparative studies assessing how various psychological therapies affect psychological capital development. Currently, two popular psychological therapies for improving mental health in various populations are dialectical behavior therapy (DBT) and group cognitive behavioral therapy (GCBT; Zhou, 2021; Yang, 2016; Zhang, 2022; Öst, 2008; Linehan, 1987; Linehan, 1993; Eisner et al., 2017; Saito et al., 2020). Research on the relative efficacy of these two treatments in boosting the psychological capital of medical students is still lacking, though. Comparative studies in medical student populations are especially crucial because of the variations in the mechanisms underlying these interventions.

This study compared the efficacy of GCBT and DBT as interventions for improving the psychological capital of medical students. In particular, we looked into whether these two programs’ effectiveness in raising medical students’ psychological capital differed significantly. After the 6-week intervention, the DBT group was projected to have considerably higher psychological capital enhancement than the GCBT group, supporting our hypothesis that DBT may be superior to GCBT in this regard. In addition to addressing the research gap in the field, this study intends to establish a scientific foundation for the use of successful psychological interventions in medical education in order to support medical students’ psychological well-being and professional flexibility. It does this by methodically contrasting these two interventions.

## 2 Research materials and methods

### 2.1 Participants

This study, which recruited sophomore undergraduate students, was carried out at a medical university between September and December 2023. Professional psychometricians were present to oversee the entire process and guarantee the uniformity, precision, and comprehensiveness of data collection. The questionnaires were distributed collectively by class at the appointed time, and students willingly participated in completing them. Based on the collection of 1,100 questionnaires, the study participants were chosen from a group of students with low psychological capital scores (the low-score group is defined as people scoring below the mean minus the standard deviation Yuan et al., 2022). It was necessary for each participant to have either normal or corrected vision. Those having a history of drug or alcohol addiction, neurological or psychiatric conditions, or those who had already received cognitive behavioral therapy (CBT) were eliminated in order to preserve the sample’s homogeneity.

Based on a statistical efficacy of 80%, a significance level of 0.05, an effect size of 0.5, and a 10–20% attrition rate, as determined by the G*Power 3.1 software, a minimum sample size of 42 people was advised. Ultimately, 65 qualified students—32 men and 33 women—were chosen to take part in the research. To ensure fairness and randomization of assignment, each participant was assigned at random to either the intervention or control groups using a computer-generated table of random numbers. An additional 18 participants were allocated to the control group, and 38 people in the intervention group were further randomized to the GCBT and DBT groups. Only the data from 56 individuals who finished the intervention were kept in the final analysis; the data from 9 participants who dropped out because of course conflicts were excluded.

Before the intervention, each participant signed an informed permission form attesting to their voluntary involvement in the research. The experiment’s precise goal was not disclosed; instead, it was only mentioned as being connected to psychological capital in order to reduce experimental bias. Social workers with specific CBT training carried out the study under the supervision of a qualified supervisor. As an observer, a psychology graduate student was in charge of gathering information and recording the intervention procedure. With the clinical trial registration number ChiCTR2400080269 and ethical review number 84230092, the study was approved by Anhui Medical University’s Ethics Committee.

### 2.2 Materials

The Positive Psychological Capital Questionnaire (PPQ), created by Zhang and Zhang (2010), was used in this study. Its 26 assessment items gauge the four primary components of psychological capital—self-efficacy, optimism, hope, and resilience—which collectively constitute a person’s positive psychological state, also known as “positive psychological capital” (Luthans et al., 2006). A seven-point Likert scale, with 1 denoting “completely disagree” and 7 denoting “completely agree,” was used to grade the participants. The total score ranged from 26 to 182, with higher scores indicating larger amounts of positive psychological capital. It is important to note that in order to properly evaluate the corresponding attributes, items 8, 10, 12, 14, and 25 of the questionnaire call for a reverse scoring approach. Prior research has demonstrated the PPQ’s strong validity and reliability among college students and other youth populations (Chen et al., 2024; Wei et al., 2024). The KMO value in this study was 0.925, while the Cronbach’s alpha coefficient of the PPQ was 0.918.

### 2.3 Methods

The study’s intervention protocols, which include the particular procedures of group cognitive behavioral therapy (GCBT) and dialectical behavioral therapy (DBT), are listed in Tables 1, 2. During the intervention phase, a weekly medical psychology course covering the fundamentals of mental health, including psychological theories and the diagnosis and treatment of mental diseases, was taken by both the intervention and control groups. The precise intervention procedures utilized in this study to guarantee the independence and scientific validity of the measured intervention effects were not included in this course, which was created to be compatible with standard medical education.

**Table 1:** The intervention goals and processes of group cognitive behavioral therapy.

Session	Objectives	Content
First	Acquaintance and Alliance, Introduction to the Cognitive Triangle	Mutual Introduction through Icebreakers; Establishing Group Rules; Setting Small Group Goals; Psychological Education: Cognitive Triangle Assigning Homework: Recording Recent Negative Emotional Events and Evaluation
Second	Identify Negative Emotions, Introduction to the Three-Column Table	Icebreaker Activity; Review Homework: Understanding Basic Emotions and Rating, Introduction to the Three-Column Table Psychological Education: Introducing Automatic Thoughts Assign Homework: Use the Three-Column Table to Record Negative Emotions and Automatic Thoughts
Third	Challenging Automatic Thoughts, Introduction to the Five-Column Table	Icebreaker Activity; Review Homework: Three-Column Table Psychological Education: How to Identify and Challenge Automatic Thoughts Assign Homework: Use the Five-Column Table to Record Negative Events, Emotions, and Thoughts, Evaluate, then Record Rational Emotional Responses and resulting Behavioral Changes
Forth	Consolidation of the Five-Column Table	Icebreaker Activity; Review Homework: Five-Column Table Psychological Education: How to Identify and Challenge Automatic Thoughts Assign Homework: Use the Five-Column Table to Record Negative Events, Emotions, and Thoughts, Evaluate, then Record Rational Emotional Responses and resulting Behavioral Changes
Fifth	Behavioral Experiment	Icebreaker Activity; Review Homework: Five-Column Table Psychological Education: How Behavior Regulates Emotions Assign Homework: Take Action Based on Distressing Emotions
Sixth	Summarize Achievements Affirm the Future, Maintain Positive Expectations	Icebreaker Activity: Review Homework: Results of Behavioral Experiment Summarize and Consolidate Members’ Learning Content, Address Separation Emotions

**Table 2:** The intervention goals and processes of dialectical behavioral therapy.

Module	Objectives	Content
Introduction to Mindfulness	1. Acquaintance and Alliance Introduction to Mindfulness	Icebreaker Activity: Getting to Know Each Other; Establishing Group Rules; Setting Group Small Goals; Psychological Education: DBT (Wise Mind); Introduction to Psychological Capital and Mindfulness Assign Homework: Mindfulness Check-In; Recording Emotional Events for the Next Week.
Emotion Regulation	2. Understanding Emotions and Assessment	Icebreaker Activity; Mindfulness; Review Homework: Mindfulness Check-In Experience; Emotional Events Psychological Education: Identifying Negative Emotions and Assessment; Introduction to the Cognitive Triangle Homework: Mindfulness Check-In; Three-Column Table
	3. Challenging Irrational Cognitions, Reverse Action	Icebreaker Activity; Mindfulness; Review Homework: Experience of Mindfulness Check-In; Three-Column Table Psychological Education: Challenging Irrational Cognitions, How to Reverse Action Homework: Mindfulness Check-In; Contrary Action Plan Table
Pain Tolerance	4. Complete Acceptance	Icebreaker Activity; Mindfulness; Review Homework: Contrary Action Plan Table Psychological Education: What is Complete Acceptance and How to Do It Homework: Practice Complete Acceptance and Fill Out Form
	5. Sensory Soothing	Icebreaker Activity; Mindfulness; Review Homework: Complete Acceptance Psychological Education: What is Sensory Soothing Homework: Practice Sensory Soothing
Interpersonal Efficacy	6. DEAR MAN Practice Summary Review, Friendly Farewell	Icebreaker Activity; Mindfulness; Review Homework: Sensory Soothing Psychological Education: How to Manage Interpersonal Relationships, DEAR MAN Practice, Maintain Mindfulness Summary Review: Summarize and Review Activities, Exchange Gifts, Address Farewell Emotions.

The six-week GCBT intervention takes place once a week on Saturdays, with two-hour sessions. The first week’s objectives were to explain cognitive triangulation theory and build relationships among group members. Following an icebreaker exercise to help members get to know one another, create group rules, and set goals, participants received psychoeducation on the Cognitive Triangle and were given an assignment to document unpleasant emotional experiences. The purpose of the second week was to help members recognize basic emotions, introduce the three-column scale, go over the assignment from the previous week, help members understand basic emotions and automated thinking, and give them a homework assignment that required them to record negative emotions and automated thinking using the three-column scale. Week three’s objectives are to introduce the five-column form and assist group members in challenging automated thinking. They will also discuss last week’s homework and provide homework that requires group members to record events, emotions, and thoughts using the five-column form in order to evaluate logical emotional reactions and behavioral changes. By revisiting assignments to strengthen mastery of the five-column form and promoting regular recording of emotional and cognitive changes, week four further reinforces the use of the form. Week five’s objective was to control emotions through behavioral experiments. After going over the assignments, group members tried to respond practically to upsetting feelings in the behavioral experiments and learned the fundamentals of behavioral regulation of emotions. In order to sustain positive expectations and describe the effectiveness of the intervention, group members summarized the behavioral experiment’s outcomes, reviewed their progress and growth during the intervention, and expressed optimistic expectations for the future in week six. Refer to Table 1.

The four primary modules of the six-week Dialectical Behavior Therapy (DBT) intervention were administered once a week on Sundays for two-hour sessions. The goal of teaching Module 1: Introduction to Mindfulness during the first week is to foster group relationships and introduce mindfulness. Group norms and objectives are established through icebreaker exercises, and psychological instruction on DBT, psychological capital, and mindfulness is given. In order to document emotional experiences for the upcoming week, participants are given homework. Weeks two and three are covered in Module 2: Emotion Regulation. Through a combination of mindfulness exercises, icebreaker games, and instruction on recognizing negative emotions, participants gain an understanding of and ability to evaluate emotions throughout the second week. Emotional events are recorded in a three-column table. The third week is dedicated to confronting illogical thinking. In addition to learning how to challenge illogical thinking, participants are exposed to the idea of “reverse action.” In addition to receiving relevant psychoeducation, participants are given the assignment of developing a reverse action plan. Weeks four and five are dedicated to Module 3: Pain Tolerance. The fourth week is all about total acceptance. Participants gain a deeper grasp of the concept of complete acceptance through psychological education, icebreaker activities, and mindfulness practice. They also perform related assignments. In order to improve pain tolerance, the fifth week’s focus is on sensory soothing. Participants engage in mindfulness exercises, study sensory calming methods, and do associated tasks. The sixth week’s Module 4: Interpersonal Efficacy focuses on enhancing participants’ interpersonal abilities. Members of the group engage in activities that handle farewell emotions, such as summarizing experiences and sharing gifts, as well as practicing the DEAR MAN approach and review tasks. The DBT intervention encourages participants to practice mindfulness exercises daily to enhance their learning. Refer to Table 2.

### 2.4 Analysis of data

SPSS 26.0 software was used for data processing and analysis in this work. The statistical significance level was set at *p* < 0.05, and effect sizes were represented using η^2^ (eta-squared) and Cohen’s d.

## 3 Results

### 3.1 Test of homogeneity for baseline attributes

Prior to the intervention, the chi-square test (*χ^2^*), the mean ± standard deviation (M ± SD) for ANOVA for normally distributed data, the H-rank-sum test (Kruskal-Wallis test) for skewed data, and the median and its upper and lower quartiles were used to evaluate baseline parameters (e.g., gender, place of birth, being an only child, being a student leader, etc.) of the participants in the three groups. to guarantee that groups’ baseline attributes are uniform. The findings demonstrated that the three groups did not differ significantly on baseline parameters (*p* > 0.05), suggesting that the distribution of the three groups by gender and place of birth was balanced (refer to Table 3). This provides a solid basis for the investigation of intervention effects that follows.

**Table 3:** Results of demographic variables and scale evaluation before intervention for three groups of participants.

Variables		Total (*n* = 56)	Control Group	Intervention Group	Intervention Group	*χ^2^/F*	*p*
			0 (*n* = 18)	1 (*n* = 19)	2 (*n* = 19)		
Gender	(M/F)	26/30	8/10	7/12	11/8	*χ^2^* = 1.99	0.37
Only child	(yes/no)	19/37	7/11	5/14	7/12	*χ^2^* = 0.88	0.645
Whether one has held a student leadership position	(yes/no)	34/22	11/7	12/7	11/8	*χ^2^* = 0.11	0.946
Whether one has performed community service	(yes/no)	49/7	15/3	17/2	17/2	-	1
Place of origin	(rural/urban)	28/28	10/8	10/9	8/11	*χ^2^* = 0.73	0.695
PPQ	Mean ± SD	87.66 ± 12.00	88.20 ± 10.57	85.05 ± 14.62	89.68 ± 10.61	*F* = 0.73	0.486
Optimistic	M (Q₁, Q₃)	22.00 (17.25, 25.00)	21.50 (18.50,25.00)	22.00 (15.00,26.00)	22.00 (18.00,23.50)	*χ^2^* = 0.10#	0.951
Hope	Mean ± SD	22.31 ± 5.32	23.35 ± 4.76	21.21 ± 5.82	22.32 ± 5.43	*F* = 0.78	0.463
Self-efficacy	M (Q₁, Q₃)	23.00 (21.00, 25.00)	22.50 (21.00,25.00)	24.00 (18.50,26.00)	23.00 (22.00,24.00)	*χ^2^* = 0.15#	0.928
Resilience	Mean ± SD	22.22 ± 4.60	21.85 ± 4.89	20.89 ± 5.23	23.95 ± 3.06	*F* = 2.29	0.111

For normally distributed data analyzed using analysis of variance (ANOVA), results are presented as mean ± standard deviation (M ± SD). For non-normally distributed data analyzed using the Kruskal-Wallis test (#), results are presented as median (upper quartile, lower quartile).‘0’ refers to the control group, ‘1’ refers to the GCBT intervention group, and ‘2’ refers to the DBT intervention group. The same applies below.

### 3.2 GCBT and DBT’s intervention effects

Significant differences between the psychological capital measures before and after the GCBT and DBT therapies are displayed in Table 4. In particular, there were parallels between GCBT and DBT therapies in terms of improving medical students’ psychological capital. With effect sizes of 0.567 and 0.667, respectively, at the medium to high effect level, both groups demonstrated a significant improvement in total psychological capital scores (*p* < 0.001), suggesting that the two interventions had comparable effects on overall psychological capital enhancement. Second, both GCBT and DBT therapies showed significant improvement (*p* < 0.05) on the optimism and hope dimensions, with similar effects and moderate effect sizes, respectively. Furthermore, the GCBT and DBT intervention groups demonstrated nearly identical improvements (*p* < 0.001) in the self-efficacy and mental toughness dimensions, with effect sizes of 0.572 and 0.556 and 0.454 and 0.572, respectively, indicating moderate to high effects. In terms of improving several aspects of medical students’ psychological capital, GCBT and DBT demonstrated remarkably similar intervention results overall. This was particularly true for self-efficacy and psychological resilience, which were nearly identical.

**Table 4:** The results of pre-and post-intervention measurements for three groups.

GCBT			*F*	*p*	*Effect size*	DBT		*F*	*p*	*Effect size*	Control group		*F*	*p*	*Effect size*
Variable [*mean* (*±SD*)]	Pre-test (*n* = 19)	Post-test (*n* = 19)				Pre-test (*n* = 19)	Post-test (*n* = 19)				Pre-test (*n* = 18)	Post-test (*n* = 18)			
PPQ	85.053 ± 14.226	105.000 ± 14.726	−4.408	<0.001	0.567	89.684 ± 10.332	109.263 ± 11.479	−5.597	<0.001	0.667	88.167 ± 10.859	96.222 ± 10.075	−2.852	0.011	0.359
Optimistic	21.105 ± 6.782	25.789 ± 6.152	−2.641	0.017	0.340	21.211 ± 5.718	25.789 ± 4.560	−3.168	0.005	0.405	20.444 ± 5.570	23.500 ± 4.810	−3.061	0.007	0.282
Hope	21.211 ± 5.662	25.316 ± 6.325	−3.137	0.006	0.324	22.316 ± 5.282	27.053 ± 4.548	−4.472	<0.001	0.433	23.833 ± 4.450	24.722 ± 4.851	−0.815	0.426	
Self-efficacy	21.842 ± 5.254	27.895 ± 3.177	−4.47	<0.001	0.572	22.211 ± 3.318	27.263 ± 4.178	−4.055	<0.001	0.556	22.222 ± 3.823	24.556 ± 3.419	−1.978	0.064	
Resilience	20.895 ± 5.088	26.000 ± 4.931	−4.034	<0.001	0.454	23.947 ± 2.982	29.158 ± 4.368	−4.729	<0.001	0.572	21.667 ± 4.922	23.444 ± 4.374	−1.868	0.079	

Additionally, there were discrepancies between the intervention effects of DBT and GCBT. Self-efficacy had the largest effect size (0.572), indicating a moderate to high intervention effect, whereas the total psychological capital score and its aspects demonstrated significant augmentation (*p* < 0.05) in the GCBT intervention group. Conversely, the DBT intervention group’s effect sizes on the optimism, hope, and psychological resilience dimensions ranged from 0.405 to 0.667, with the total psychological capital score having the largest effect size at 0.667. The DBT group’s effect sizes on these dimensions were marginally higher than those of the GCBT group, at 0.405, 0.433, and 0.572, respectively. The GCBT group’s effect size (0.572) was greater than the DBT group’s (0.556) on the self-efficacy component, indicating a stronger intervention effect. This suggests that each of the two interventions had its own strengths in different psychological capital dimensions. For example, DBT was marginally more effective than GCBT in enhancing the total psychological capital score, optimism, hope, and psychological resilience, while GCBT had a more significant effect on the self-efficacy dimensions.

### 3.3 The control group’s effects

Only the total psychological capital score and the optimism dimensions (effect sizes of 0.359 and 0.282, respectively) showed significant improvement (*p* < 0.05) in the control group; no significant differences were found in the other dimensions of psychological resilience, hope, or self-efficacy (*p* > 0.05). This implies that there was little change in the control group and that psychological capital was only marginally impacted by regular curriculum-based schooling.

### 3.4 Results of an ANOVA

Following the intervention, the ANOVA results for the three groups are shown in Table 5. There was a significant difference between the three groups, according to the ANOVA results for the total psychological capital score (*F* = 4.31, *p* = 0.018). Subsequent post-hoc analyses revealed significant differences between the control and DBT groups (*p* = 0.003) and between the control and GCBT groups (*p* = 0.039, effect size η^2^ = 0.161). Significant between-group differences were also found by the ANOVA for the mental toughness measure (*F* = 7.70, *p* = 0.001). The greater intervention effect of DBT on this dimension was further supported by the substantial differences in mental toughness between the GCBT and DBT groups (*p* = 0.043, effect size η^2^ = 0.206) and between the control and DBT groups (*p* = 0.001).

**Table 5:** Post-test variance analysis for the three groups.

Variables *(Mean ± SD)*	Total (*n* = 56)	Control group (0) (*n* = 18)	GCBT (1) (*n* = 19)	DBT (2) (*n* = 19)	*F*	*p*	Post hoc pairwise comparisons *p*	*Effect* *size*
PPQ	103.83 ± 13.35	97.55 ± 10.65a	105.00 ± 15.13b	109.26 ± 11.79b	*F* = 4.31	0.018	(0–1) 0.039	0.161
							(0–2) 0.003	
Optimistic	25.16 ± 5.33	23.95 ± 4.93	25.79 ± 6.32	25.79 ± 4.69	*F* = 0.77	0.466		
Hope	25.83 ± 5.44	25.15 ± 5.07	25.32 ± 6.50	27.05 ± 4.67	*F* = 0.71	0.494		
Self-efficacy	26.72 ± 3.93	25.10 ± 3.78	27.89 ± 3.26	27.26 ± 4.29	*F* = 2.92	0.063		
Resilience	26.12 ± 5.14	23.35 ± 4.30a	26.00 ± 5.07a	29.16 ± 4.49b	*F* = 7.70	0.001	(0–2) 0.001	
							(1–2) 0.043	0.206

**Note: “0” represents the control group, “1” represents the GCBT group, and “2” represents the DBT group.**

## 4 Discussion

The impact of GCBT and DBT in raising medical students’ psychological capital was examined in this study. The study’s findings indicate that while both can successfully improve medical students’ psychological capital, there are some minor variations in particular areas, which may be due to the two interventions’ different theoretical underpinnings and technical foci: DBT focuses more on emotional regulation, pain tolerance, and positive thinking exercises to help people find a balance between stress and emotional swings, while GCBT primarily modifies negative thinking and behavioral responses through cognitive restructuring and behavioral activation (Mehta, 2018; Sehati et al., 2019). In addition to offering theoretical backing for medical students’ psychological capital intervention techniques, this research offers recommendations on how to successfully raise medical students’ psychological capital in the real world (Heikkila et al., 2024; Zhou, 2021).

### 4.1 Similarities between the outcomes of GCBT and DBT interventions

When it came to improving medical students’ psychological capital and its aspects, GCBT and DBT had striking similarities. This could be because both interventions are grounded in the cognitive-behavioral theoretical framework, which improves medical students’ psychological capital through behavioral and cognitive changes, leading to comparable benefits in raising self-efficacy, optimism, and hope (Yu et al., 2023). This is in line with earlier studies that have demonstrated the substantial benefits of CBT-based intervention strategies in fostering psychological capital and wellbeing (Heikkila et al., 2024). Furthermore, both DBT and GCBT employ a group intervention structure to give medical students social networks and emotional support, which in turn improves a sense of community and social support and fortifies psychological resilience and self-confidence (Bryde Christensen et al., 2021). In addition to fostering connections of mutual support, this group support environment was crucial in helping medical students build their psychological capital. As a result, when it came to improving medical students’ psychological capital and its aspects, GCBT and DBT showed very comparable results.

### 4.2 Differences between GCBT and DBT therapies’ efficacy

Despite the fact that GCBT and DBT had comparable effects on a number of dimensions, this study found that DBT was marginally more effective than GCBT in terms of optimism, psychological resilience, and hope. This could be because DBT is better at managing emotions and handling high-pressure situations; it improves psychological resilience and optimism by strengthening emotion regulation skills, which help medical students stay optimistic when faced with academic stress (Rabiee et al., 2020; Gillespie et al., 2022). Additionally, medical students receive thorough training in psychological skills through the multi-module integration of DBT (such as emotion regulation, positive thinking, distress tolerance, and interpersonal efficacy), which shows a notable advantage in mental toughness in particular (Ji, 2018). Furthermore, DBT is better than GCBT’s cognitive restructuring technique in the hope component because its dialectical thinking training and reality acceptance techniques support medical students in retaining optimism and constructive coping mechanisms in trying circumstances (Alfonsson et al., 2022). While DBT focuses more on emotion regulation (Li et al., 2022), it is less effective than GCBT at directly boosting self-efficacy. Instead, GCBT’s superiority on self-efficacy may be due to its emphasis on goal setting, cognitive restructuring, and problem solving (Menefee et al., 2022), which helps medical students improve their sense of accomplishment and self-confidence by setting specific goals and adjusting negative self-evaluations. Overall, GCBT was better at increasing self-efficacy, whereas DBT was marginally better at increasing optimism, mental toughness, and hope. Each was successful in a distinct dimension.

### 4.3 Study limitations and suggestions for more research

There are certain limitations, even though the current study offered insightful information about how GCBT and DBT might be used to improve medical students’ psychological capital. First, the evaluation of the intervention’s long-term benefits was limited by the study’s brief one-month follow-up period. To get more thorough information on the persistence of the intervention, future research could increase the follow-up duration to 3 months or more. Second, the results may not be as broadly applicable as they may be due to the limited sample size. To improve representativeness, future research should extend the sample size to include medical students from a range of socioeconomic backgrounds, genders, and grades. Furthermore, this study did not perform subgroup analyses of demographic characteristics (e.g., gender, years of study) on the impact of the intervention (Simmonds-Buckley et al., 2021). Since the sample size allows for a more thorough examination of the variations in how medical students with various characteristics respond to GCBT and DBT, future research may employ subgroup analysis. Given that all of the data in this study were self-reported by medical students, response bias may have occurred. Subjects may have selected more favorable answers because of social pressure or expectation of the intervention, which would have produced results that were not entirely accurate. To further evaluate the effects of the intervention and lessen response bias from self-report, future research might think about adding objective assessment instruments or observational data (such as eye movements). In order to guarantee the applicability and generalizability of the intervention program in a wider context, future research should take into account the possible influence of cultural variations on intervention effects, as this study was carried out in a particular cultural setting.

### 4.4 Conclusion and practical application

The findings of this study show that both GCBT and DBT may significantly improve a number of dimensions and effectively raise medical students’ psychological capital. In light of this, it is advised that DBT interventions be prioritized in medical students’ mental health training. This can be done by establishing a special DBT group within the curriculum or by scheduling a DBT module in adaptive training at the start of each semester. The multi-module design of DBT (such as emotion regulation and pain tolerance) is especially well-suited for the psychological needs of medical students in high-pressure settings. For instance, it can be used to teach emotion management skills in the classroom and provide group support during the clerkship or exam season.

Furthermore, as a cognitive intervention technique, GCBT can be utilized in conjunction with DBT and has a remarkable impact on raising medical students’ self-efficacy. By establishing clear objectives and modifying self-evaluation, GCBT modules can be incorporated into career development courses or skills workshops to assist medical students in improving their sense of accomplishment and self-efficacy. In the future, group DBT and GCBT can be combined in medical students’ mental health education to offer all-encompassing psychological support in both group and classroom settings. This will progressively build medical students’ psychological capital, establishing a strong basis for their mental health and professional growth.

## Data Availability

The datasets presented in this article are not readily available because access to the dataset is restricted to authorized research personnel only. The dataset is intended solely for non-commercial academic research purposes, and any sharing or redistribution of the data is strictly prohibited without prior authorization. Requests to access the datasets should be directed to Cexin Dong 694709111@qq.com.
